# Exploring the Associations of Walking Behavior with Neighborhood Environments by Different Life Stages: A Cross-Sectional Study in a Smaller Chinese City

**DOI:** 10.3390/ijerph17010237

**Published:** 2019-12-28

**Authors:** Ziwen Sun, Ka Yan Lai, Simon Bell, Iain Scott, Xiaomeng Zhang

**Affiliations:** 1 OPENspace Research Centre, Edinburgh School of Architecture and Landscape Architecture, The University of Edinburgh, Edinburgh EH1 2LE, UK; s.bell@ed.ac.uk (S.B.); iain.scott@ed.ac.uk (I.S.); 2 Healthy High Density Cities Lab, HKUrbanLab, The University of Hong Kong, Pok Fu Lam, Hong Kong, China; laikyy@hku.hk; 3 Department of Landscape Architecture, Estonian University of Life Sciences, 51014 Tartu, Estonia; 4 Centre for Global Health, Usher Institute, The University of Edinburgh, Edinburgh EH8 9AG, UK; xiaomeng.zhang@ed.ac.uk

**Keywords:** walkability, age-spatial separation, everyday life, neighborhood environment, smaller Chinese cities

## Abstract

Because of high population density and rapid urbanization, different human life stages have distinct growth experiences, leading to different lifestyles and age-spatial separation in the same neighborhood environment, particularly in smaller Chinese cities. The relationship of environment to physical activity may differ from western or larger Chinese cities. This study examined the associations of walking duration to the neighborhood environment and other factors, and explored the nuances of walking behavior for different life stages of adults in a smaller Chinese city, Yuncheng. An interviewer-administered questionnaire survey (*n* = 173) and face-to-face interviews (*n* = 19) were conducted in August 2017. Descriptive analysis and multiple linear regression were performed to describe walking motivations, sociodemographic characteristics, neighborhood environments, and their impacts on walking duration across three life stages. The quantitative findings were followed by interviews to validate and interpret them. Our results showed no positive associations of land-use mix (LUM) and residential density on walking duration, and even inverse associations of LUM-recreation and LUM-education for specific life stages were identified. Younger people’s walking behavior was more related to consumption amenities distinct from those of older people. Our findings suggest that using walkable neighborhood policies (e.g., high residential density and land-use mix) to increase physical activity might be ineffective in smaller Chinese cities.

## 1. Introduction

Being physically inactive in contemporary cities is resulting in more than 5 million deaths annually worldwide through its impacts on non-communicable diseases [1]. With the shift of healthcare strategies from treatment to prevention, the built environment has been taken as a potential form of intervention to enhance physical activity [2]. Much of the existing research indicates that adults tend to be more physically active if they live in high-density and mixed-use neighborhoods, with a range of functional amenities within walking distance [3,4,5,6,7,8]. Of all types of physical activity, walking is the most common in everyday life across most population groups, regardless of age, social class, race or gender, with minimum skills, infrastructure, or equipment required [9,10,11].

Similar concerns and negative consequences regarding declining walkability have also emerged in Chinese cities since China’s economic reforms in the late 1970s [12,13,14]. For instance, the prevalence of obesity increased from 8.6% in 2000 to 12.9% in 2014, leading to increasing risks for non-communicable diseases [15,16]. Although many Chinese scholars have acknowledged problems arising from rapid urbanization and urban expansion, most of them have focused on social differentiation, equality and justice [17,18,19,20]. In recent years, a few studies in relation to walkability have been conducted in large Chinese cities, such as Shanghai [14,21], Shenzhen [22], Hong Kong [13,23,24,25], Xi’an [26], and Hangzhou [14]. Some inconsistent findings and even contrary conclusions have been found in the Chinese context. There is an absence of research on how neighborhood walkability is considered in smaller Chinese cities.

According to China’s latest census data in 2010, only 26.7% of the population was living in large cities (i.e., the 30 cities that had more than 8 million residents) [27]. Large cities, mostly flooded with highly educated people, are usually connected to rapid economic growth and engage heterogeneous migrants from other areas with complex everyday lives. People living in large cities with extensive public transport infrastructure spend less sedentary time, compared with those living in smaller cities [11]. A few recent studies compared different Chinese cities and found that smaller cities in general also suffered the most from sprawl [28,29]. Smaller cities, where most Chinese people spend their lives, seem less important and have attracted less attention with few studies on them. Most concepts and policies regarding walkability that are applied are conceived either to fit the situation of larger cities or to emulate western urban models [30,31]. There is a lack of empirical evidence to clarify context-specific walking behavior in smaller Chinese cities.

Bourdieu (1984) proposes the term habitus and notes that it can guide people to think, feel and act in specific ways [32]. Different habitus with the local context (e.g., socially, economically and culturally) in developing countries may lead to different walking motivations and behaviors distinct from western ones [13,25,31,33,34]. Moreover, as found by Travlou (2006) and Ward Thompson (2007), playing in a natural environment in childhood was found to be an important factor for engagement in outdoor environments in later life [35,36]. Living in socially disadvantaged neighborhoods during childhood was found still to have negative effects on the activity levels of people aged around 70 [37]. In light of the unprecedented development and transformation of contemporary China, various growth experiences at different life stages may shape age-related habitus and distinct walking behaviors. It is vital to examine how the associations of the neighborhood environment and walking behaviors differ among people at different life stages in Chinese cities of all sizes, not just on the large ones. 

The purpose of this study, therefore, is to examine the associations of walking duration with the neighborhood environment in a smaller Chinese city, Yuncheng. We further explore nuanced associations by different life stages and interpret the statistical findings by interviewing residents, so as to demonstrate the value of bringing local everyday lives into in-depth understanding. We believe this is crucial for informing integrated physical activity policies, so that future recommendations can be made based on people’s “real life”. Based upon the above literature review, the following two research questions are presented:

- What are the associations between walking duration and neighborhood environment attributes as well as other factors? Are those associations similar to other studies? And why?
- Do the associations vary among different life stages? And if so, why?

## 2. Methods

### 2.1. Study Area 

Yuncheng is a typical smaller Chinese city located in the southernmost part of Shanxi province of central China, with a land area of 14,183 km^2^ and 5.28 million population. The administrative division of prefecture-level cities in China includes municipal districts and other units, including county-level cities, counties, and towns. Prefecture-level Chinese cities such as Yuncheng are often similar to Western cities in many studies [29]. As a typical smaller Chinese city, Yuncheng only has one municipal district (named Yanhu) with a number of county-level cities and counties. Due to the fact that other units usually include some non-urbanized areas, our study focuses on the central urban area, Yanhu district. In 2016, the population in the district was 0.68 million with a gross population density of 560 people per km^2^ and a 7.8% increase in average annual rate of GDP (gross domestic product) [38]. 

In the officially-classified group, Yuncheng belongs to small- and medium-sized Chinese cities. According to China Business Network Co., Ltd. (2017), experts recently evaluated and ranked 338 Chinese cities on 5 tiers (i.e., commercial resource agglomeration, connection with other cities, citizen vitality, lifestyle diversity, and future plasticity) [39]. Yuncheng belongs to Tier 4, which means that the city is smaller and of a type that is commonplace in contemporary China. The term “smaller city” is relative to the Chinese context and in many European countries such a size would not be considered small.

### 2.2. Population

Globally, a growing number of studies have focused on children and older people, due to the differentiation in their physiology and perception [35,36,40,41,42,43,44]. We acknowledge that studies of older people and children are crucial for walkability, but this study focuses on the “working age population” (i.e., those aged between 18 and 59) in order to avoid the potential effects of physiological differentiation upon the exposure-outcome relationship. In contemporary Chinese cities, life experiences, such as ways of working, living and spending leisure time, differ enormously across different life stages, in ways that co-produce a tangled system of complex everyday activity patterns in the same neighborhood environment. 

During the post-1949 socialist era in China, Mao’s egalitarian policies [45] meant that people who worked for the same state-owned enterprise lived together, regardless of their working position, forming what was called a work unit housing system [46,47]. Most units provided an integrated neighborhood environment of work, residence, and services (i.e., land-use mix) [21], as a walkable and sociable neighborhood. Those who experienced this dramatic transformation of the built environment and the huge shift in everyday life in their childhood, are now aged between 50 and 60. The college entrance examination was reinstated in 1977, which led to lower educational attainments in people who were over 40 during the time of our research. In the late 1990s, after the reform, people with higher incomes tended to move out, and the work units were less well-maintained and gradually decreased in number [21,26]

According to the understanding above and the age groups used in a study of socio-spatial segregation in China [48], the “working age population” was divided into three life stages (TLS): late adolescents (aged 18–25), young adults (aged 26–35), and middle-aged adults (aged 36–59). The classification of TLS was discussed and refined twice with the local participants:

- The late adolescents group has a better experience growing up, in a high-quality built environment. The legal age of majority is 18, and this is an age at which people are generally pursuing undergraduate study.
- The young adults group experienced the rapid change in the built environment after the Chinese reforms in the late 1970s. This age group starts at 26, which is often identified as the age at which there is a change from student status, and people move to having stable jobs and starting a family [20].
- The middle-aged adults group represents the generation who grew up at the time of the Chinese reform, but they have not yet reached official retirement age.

### 2.3. Questionnaire Design and Sampling

The Neighborhood Environment Walkability Scale-Abbreviated version (NEWS-A) is one of the most commonly used self-evaluation questionnaires aiming at measuring people’s perceived neighborhood environmental attributes related to physical activity. It was initially applied in developed countries, including the USA and Australia [49,50], and has been widely validated in different countries and cities [51,52,53,54]. The pilot study we first undertook initially adapted the NEWS-A questionnaire on the basis of literature review and on-site observations. Following this, broad discussion with experts and local residents reduced and adapted all the items of particular relevance to Chinese adults, ensuring the applicability or ease of use by all age groups. The 54 neighborhood characteristic items [23,24] were increased to 74, and then reduced to 59, including additional items of walking duration, walking motivations, and sociodemographic characteristics [8,55].

During the pilot study in January 2017, several practical problems were identified in the smaller Chinese city: (1) due to lower education levels and less research participation experience, many participants were less rigorous, and they always tried to guess or assume what the right answers should be, rather than expressing their real experiences and personal thought; (2) most participants who were encountered in the street did not have the patience or time to complete the questionnaire fully; (3) some questionnaire items were less connected to participants’ daily lives (e.g., street slope) and these needed to be reduced or clarified in relation to the immediate surrounding environment.

In order to overcome these problems, seven trained volunteers from Yuncheng University were recruited to implement the interviewer-administered questionnaire survey in August 2017. This period was chosen since people generally spend more time outdoors during warmer weather. It was conducted in central urban streets in order to increase opportunities to recruit more participants randomly and to collect mixed views of the different life stages. During the formal surveys, volunteers only explained the items and avoided affecting participants’ judgments; each participant usually spent less than 30 min filling in the questionnaire. The balance of genders and TLS was controlled. After completing the survey, each participant was given a non-monetary incentive as a token of appreciation for their contribution to the research. As this study is an initial exploration of smaller Chinese cities and the sample size of most studies investigating the associations between the built environment and physical activity was 101–300 [56], we collected 200 questionnaires. All data was carefully checked in three steps of screening, diagnosis, and editing [57] and 27 questionnaires were excluded. This study was approved by the ECA Ethics Committee of the University of Edinburgh (06032017).

### 2.4. Interviews

Face-to-face interviews were separately conducted by the main researcher proficient in Mandarin and local dialects. The balance of genders and TLS was controlled for the participants of interviews (*n* = 19) and there was no overlap with participants of the questionnaire survey. Before the interview, all the interviewees provided a written informed consent on participating in this research project. The interview questions were based on the NEWS-A items and the quantitative results, in order to validate and interpret unexpected findings [30]. Photos and explanations were provided if participants encountered difficulties on understanding certain questions. Participants were allowed to talk openly and to diverge from the content and theme at times. As most participants were reluctant to have their voice recorded, notes were taken during the interviews and then expanded into transcripts immediately after the conversation.

## 3. Measurement

### 3.1. Walking Duration

“Walking duration”, the outcome variable, was assessed by a single item. Participants were asked to rate “How much time do you spend on walking per day?” by using a 5-point scale (1 = 10 min or below; 2 = 11–30 min; 3 = 31–60 min; 4 = 61–120 min; 5 = 121 min or above). Based on the results from the pilot study, below 10 min and above 60 min were identified as rare cases, although a few participants who loved sports reported over 120 minutes’ walk per day.

### 3.2. Potential ‘Walking Duration’-Related Variables

“Walking motivation” includes 7 items measured by a 5-point scale (e.g., “to office”, “to school”, “to Internet bar”, “to chat/poker/mahjong”, “to stroll/exercise”, “to shop/dining” and “walking with children”). “Walking preference” was measured by rating a single item (i.e., Do you like walking?) on a 3-point scale (1 = dislike; 2 = normal; 3 = like).

“Sociodemographic characteristics” include gender, educational attainment, occupation, income, and household ownership of transportation. Educational attainment was divided into three categories: higher school or below, junior college, and bachelor or above. Occupation was divided into four categories: state workers, corporate workers, self-employees, and others. Monthly income was divided into four categories: 3000 Yuan or below, 3001–5000 Yuan, 5001–10,000 Yuan, and 10,001 Yuan or above. Participants were asked about the number of bicycles, motorcycles and cars owned per household, as well as whether they were living with children or not.

“Environmental characteristics” include 33 items with 3 subscales. The first subscale of residential density, comprising 6 items, was rated on a 5-point scale (1 = none; 2 = a few; 3 = some; 4 = most; 5 = all) and calculated by a modified scoring method [58]. A higher score indicates a higher level of residential density. The second subscale of land-use mix was assessed via a 5-point scale (1 = 30 min; 2 = 21–30 min; 3 = 11–20 min; 4 = 6–10 min; 5 = 1–5 min) to measure participants’ walking duration of accessing 20 amenities. A higher score indicates a higher level of land-use mix. The third subscale of neighborhood quality was rated using a 4-point Likert scale (ranged from 1 = strongly disagree to 4 = strongly agree).

### 3.3. Factor Analysis for Identifying Dimensions

Factor analysis was conducted to identify dimensions of independent variables and covariates. Principal component analysis with the rotation method of Varimax with Kaiser Normalization was performed. Items with factor loadings lower than 0.50 were excluded. All the subscales below have Kaiser–Meyer–Olkin (KMO) scores higher than 0.50 as a bare minimum, indicating acceptable levels of sampling adequacy [59,60]. All the dimensions with more than one item have Cronbach’s Alpha scores higher than 0.50 at significant levels (*p* = 0.000), indicating accepting levels of internal consistency. The results of factor analysis were described in the supplementary materials.

Walking motivation (Table S1): 7 items were divided into four dimensions: (a) child walking with 2 items (i.e., walking with children and walking to school); (b) recreational walking with 2 items (i.e., walking to play and walking for exercise); (c) walking to work as a single item, including walking to bus stations; and (d) social walking as a single item (i.e., walking for shopping or dinner). In smaller Chinese cities, shopping or having dinner is common social activities and often involving several friends together.

Environmental characteristics: 7 items of neighborhood quality were divided into three dimensions (Table S2): (a) social quality with 3 items (i.e., trees, interesting things, and familiar people); (b) street quality with 3 items (i.e., thrown rubbish, car parking, and building aesthetics); and (c) safety issues at night as a single item. 20 items of walking distance of land-use mix (LUM) were divided into four dimensions (Table S3): (a) LUM-daily essential with 7 items (e.g., restaurant and supermarket); (b) LUM-recreation with 5 items (e.g., karaoke bar, bath center/spa, and Internet bar); (c) LUM-education with 2 items (i.e., other schools and book stores); and (d) LUM-service with 2 items (i.e., library and post office).

### 3.4. Analysis

Descriptive analysis was conducted to present percentage, mean, and standard deviation (Table 1 and Table 2). By performing Chi-square test or one-way ANOVA, the *p*-value lower than 0.05 indicates the differences across TLS, to respond to the hypothesis of different life stages having different everyday lives and demands. Both bivariate analysis and multiple linear regression were conducted across TLS. Bivariate analysis was conducted to identify the correlations between walking duration, walking preference, walking motivation, and environmental characteristics (Table S4). Only the significant variables were further included in the multiple linear regression, which was performed to compare the three models and the associations between walking duration and the neighborhood environment and other factors (Table 3). Model 1 included variables of sociodemographic characteristics. Model 2 further included variables of walking preference and walking motivation. Model 3 additionally included variables of environmental characteristics. All statistical analyses were performed on IBM SPSS version 23 (Armonk, NY, USA) and the thresholds for *p*-value were set at 0.05.

The principal findings of the quantitative analysis were extracted into simple questions or themes, which were used for interviewing the participants. All findings obtained from the survey and interviews were combined and compared with facilitate discussion, adding nuanced and in-depth understanding of the complex meanings of neighborhood environments and walking behaviors across TLS.

## 4. Results

### 4.1. Descriptive Analysis

Table 1 shows that all participants were equally distributed across TLS and male participants accounted for 52.6%. About half of participants were living with children. The distribution of occupation and living with children for late adolescents were distinct from other two groups. Middle-aged adults tended to have lower educational attainments (e.g., 31.6% had graduated from high school or lower) but higher monthly income, compared to late adolescents and young adults. Table 2 illustrates that the average daily walking duration for all participants almost reached 31–60 min (mean = 2.95) and middle-aged adults walked even more (mean = 3.23). The walking motivation was mainly for social reasons (mean = 2.85) while with a child was the least common (mean = 2.19). The average LUM-all (20 amenities) was around 11–20 minutes’ walk (mean = 3.19). LUM-daily essential was the shortest (mean = 3.72) while LUM-service was the longest (mean = 2.13).

In short, the results of descriptive analysis demonstrate statistically significant differences among TLS, consisting of (a) living with children, education level, occupation, and income; (b) walking duration, child walking, and social walking; and (c) LUM-all, LUM-daily essential, LUM-recreation and LUM-education.

### 4.2. Multiple Linear Regression

Table S4 in supplementary materials shows the results of bivariate analysis to identify the potential variables for the multivariate analysis. Finally, walking preference, recreational walking, child walking, walking to work, LUM-recreation, LUM-education, and social quality were retained for further analysis. Using the multiple linear regression, Table 3 demonstrates the associations of walking duration with sociodemographic characteristics, walking preference, walking motivations, and environmental characteristics in three models. Regardless of the pooled data or TLS, an increased adjusted R square was found from Model 1 to Model 3, suggesting a better explanatory power of Model 3 to the outcome variable. In general, walking duration in Model 3 was positively associated with recreational walking and social quality, but negatively associated with junior college, self-employees, and LUM-recreation.

Among TLS in Model 3, walking duration of late adolescents was positively associated with recreational walking, but negatively associated with self-employees, living with children and LUM-recreation. Walking duration of young adults was positively associated with walking to work and social quality, but negatively associated with state workers, corporate workers, self-employees and LUM-recreation. Walking duration of middle-aged adults was positively associated with child walking, but negatively associated with living with children and LUM-education.

## 5. Discussion

### 5.1. Negative or Non-Impact of Land Use

Many studies from western countries showed that physical activity was positively related to high residential density and land-use mix [3,4,5,6,7,8]. However, our study found four contrary results: LUM-recreation and LUM-education were negatively associated with walking duration; there were no significant associations with LUM-daily essential and population density. Is the strategy of developing a high-density neighborhood with land-use mix inefficient to increasing physical activity in smaller Chinese cities, such as Yuncheng? A few studies from non-western countries also found similar results. For instance, Lu et al. (2017) found that land-use mix and street connectivity were not significantly associated with any domains of walking while higher neighborhood density was negatively associated with recreational walking in Hong Kong [13]. Gomez et al. (2010) proved no significant associations of land use or population density with physical activity in Bogota [33]. Via face to face interviews, the latent meanings of these contrary results were further uncovered in the local context.

**Figure 1:**
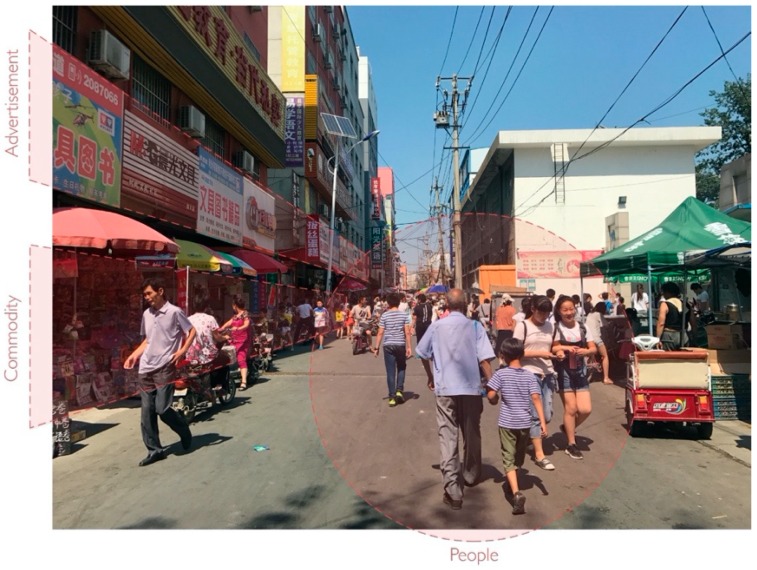
Age-related spatial segregation for LUM-education: children behaviors, demands and supplies of shops, and advertisements.

As reported by the interviewees, most parents have to work during the day, so taking care of children becomes a commercial opportunity. This results in many and varied educational amenities (e.g., after-school homework tutoring, and the teaching of special skills, such as dance and drawing), as well as other amenities for children (e.g., selling toys, stationery, special snacks, and sweet drinks) emerging around schools. Considering the features related to children (e.g., safety issues and limited walking territory), these amenities need to be as close to schools as possible [34,43]. Their type and opening times relate to schools’ agendas and aim to meet children’s demands in various ways, instead of satisfying adults’ everyday demands (Figure 1). The neighborhood environment is significantly transformed by the schools’ affiliations over time into a specific type of neighborhood, geared specially for the after-school market and of little value to anyone else. As older adults are more sensitive and likely to be affected by the neighborhood environment [40,41,42], the educational amenities, therefore, negatively impact on walking duration of middle-aged adults in our study.

The interviews pointed the way to understanding these recreational amenities (e.g., karaoke bar and cinema) as extremely important for irregular social activities, rather than for basic everyday demands or itinerant individual activities. People prefer to go to these amenities together, from different parts of the city, and the amenities generally have variously sized spaces inside, so that different numbers of friends, family and colleagues can interact when “doing business”, “strengthening the feeling of contact” and “socializing”, as with the recreational amenity of massage [17]. Moreover, these recreational amenities have higher profits and tend to be located together as a business strategy to facilitate competitiveness. This transforms the surrounding environment into a semi-isolated area for adults’ irregular recreational time (i.e., holidays, weekends, or nights), as per the capacity of schools’ space-production [34]. In other words, the neighborhood environment is not suitable for daily living and regular needs, and the association is likely to be negative for walking duration.

Land-use mix and residential density are generally considered as key determinants for walkability in western countries [6,10,14]. Unexpectedly, they were not associated with walking duration in this study. Particularly “LUM-daily essential” (e.g., restaurant and supermarket) should be closely related to everyday demands and walking duration in our hypothesis. Participants noted that most of the daily amenity items (e.g., street vending, restaurant, laundry, and salon) were considered as “sure-fire returns” and “less investment” to meet necessary and basic everyday demands. Specifically based on the Chinese context with high population density and rapid urbanization, if any neighborhood lacks these daily amenities, people consider seizing the great opportunities promptly. For example, street vending as a flexible mobile amenity timely meet people’s everyday demands, even in peri-urban areas [12]. Therefore, no matter whether walking duration is higher or lower, most neighborhoods usually include numerous daily essential amenities which originate as organic and traditional developments. This suggests that though land-use mix (particularly LUM-daily essential) and high residential density are likely associated with high walkability, they may be not an issue in smaller Chinese cities.

### 5.2. Three Life Stages

We found that walking duration in general was positively associated with recreational walking and social quality (i.e., seeing other people or activities), but negatively associated with disadvantaged groups (e.g., junior college students and self-employees), in line with some findings from previous studies in China [13,24,25,30,48]. In our study, the associations are further discussed across TLS.

Late adolescents’ walking duration was only positively associated with recreational walking (i.e., walking to play and walking to stroll). People aged 18–25 in contemporary China are possibly studying at university and living together with six to eight roommates, leading to distinct walking behaviors. Most campuses in Yuncheng have been relocated outside the city center to take advantage of lower land prices, vacant plots and to cater for a large number of students. The land can be independently developed as work units [46,47], providing an integrated environment of study, residence and daily services in an otherwise isolated area. The surrounding environment has evolved closely in line with the universities’ agendas and students’ specific recreational demands, such as consumption amenities (e.g., Internet bars, cheap hotels, and small restaurants). Moreover, younger people are likely to be concerned about social interaction rather than environmental quality [35,36]. An adolescent participant noted they had similar schedules to those of their roommates, and therefore often walked with them to study, play and eat:

“Playing computer games together can build a closer relationship with your roommates, otherwise you may be isolated from others. We always ask each other to eat, play, or study together [which increases walking]. All our everyday activities can be met within the surrounding neighborhood [a walking distance].”

Young adults’ walking duration was associated with various items. People older than 26 usually have a formal job and are married, in contrast to the adolescent lifestyle [20,44]. Table 1 shows that young adults (61.8%) and middle-aged adults (77.2%) were more likely to live with children, compared with late adolescents (12.5%). Ding et al. (2011) found that Chinese adults who were married were less active. This may explain why the average walking duration of young adults was the shortest in this study (mean = 2.79) compared to the other two life stages [30]. In addition, participants noted that most young adults reached the average income level (Table 1), but the disadvantaged groups had to sacrifice their leisure time to work harder and earn more money, resulting in feeling tired and being less active during their rest or recreational period, as also found in a study of immigrants and social-spatial separation in Weizhou [48]. For example, a 27-year-old participant said:

“After studying, I suddenly realized the differences of family class. My previous classmates who have powerful parents found good jobs or businesses. However, I have to depend on myself and work hard. I do not have time to do recreational walking and sometimes I have to walk [to work] to save money.”

**Figure 2:**
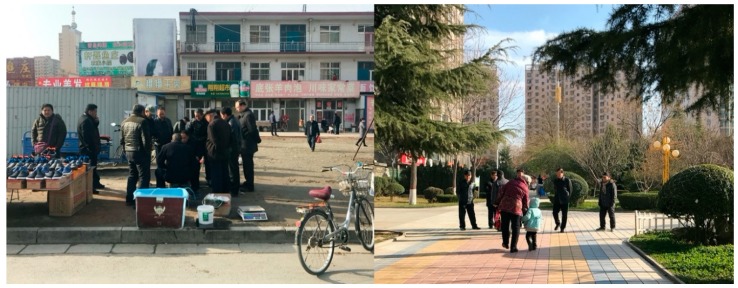
Older people’s self-created space and government-financed “free” amenities.

Middle-aged adults’ walking duration was positively associated with child walking but negatively associated with LUM-education. The older people in our study generally had lower educational attainments (Table 1), suggesting a different classification for advantaged/disadvantaged groups compared to other two life stages. Furthermore, middle-aged adults were ambivalent about consumption, possibly owing to their experience growing up (e.g., egalitarian policy and collectivism in state-owned enterprises) [21,45,46,47], revealing that the same amenities have different degrees of attraction to different life stages. Older people are willing to spend money to provide a higher-quality growth environment for their children, but their daily consumption is extremely low. Most older participants in our study prefer to use basic types of amenity for their everyday life as “survival” and other types of free amenity for themselves as “enjoyment” (Figure 2). As such, the preferred walkable neighborhood at this life stage is mainly related to basic (e.g., cheap local stores) and free amenities (e.g., squares and parks). According to a male participant (aged 52):

“I often play [Chinese chess] on the street and eat at home. The aim of our generation is to earn more money for our children’s education. Low education means you [younger people] have to do heavy and dirty labor works.”

In general, younger people perceived a shorter walking distance to various amenities, and older people had higher income and lower educational attainment. This suggests that the former results from human nature, but the latter is likely to differ in the future and therefore needs to be continually studied in smaller Chinese cities. In terms of walking motivation, there was a clear differentiation across younger to older people: from self to family. Younger people were more concerned about social interaction and more likely to be attracted by consumption amenities, while older people tended to be less involved in consumption in their everyday lives. This uncovers the fact that various individual-financed amenities within a walking distance may increase physical activity of younger people, while social events and free government-financed amenities are likely to increase the physical activity of older people.

### 5.3. Smaller Chinese Cities and Development Patterns

Unlike small cities, large cities often have multiple modes of transportation and extensive transport infrastructure to facilitate their residents, leading to less sedentary time [11]. There are no studies that compare the associations of walking behaviors between the bigger cities and smaller cities in China. A few recent studies examined urban sprawl across all Chinese cities and made a surprising finding that smaller cities generally sprawled more compared to bigger cities—potentially making them less walkable [29]. One reason is that smaller cities are usually overlooked by the central government due to their lowly position and the large number, and local authorities impact more on their temporary urban development, compared to large cities. Meanwhile, the central government also encourages the improved development of smaller cities [29]. This might lead to urban sprawl, but also provide a chance for organic and traditional development. In other words, the built environment in smaller Chinese cities is developed more based on the real-time demands of the residents and there is less control for temporary informal practices, such as street vending [31]. The findings in our study, therefore, may differ from those in large Chinese cities or Western countries.

## 6. Conclusions

In contemporary China, due to rapid urbanization, the built environment and lifestyles are changing much faster than in developed countries. The critical aspect is that different life stages underwent different experiences growing up, leading to different everyday demands and thereby producing age-related differences and socio-spatial separations. For example, a series of amenities (e.g., cinema, Internet bar and café) that tend to be used by younger adults and rarely by older adults, have produced age-related walking behaviors. Namely, compared with older people, younger people’s walking behavior may be more related to land-use mix, although our quantitative results showed no association. Their choices of work, shopping areas and leisure activities are in some ways distinct from those of older people, producing nuanced associations of walking/physical activity to the same neighborhood environment in smaller Chinese cities. Over time, LUM-education or LUM-recreation might reduce the degree of mixed-use neighborhoods and transform the surrounding environment to meet the specific demands of students or irregular socio-playing engagements, which were negatively associated with the walking/physical activity of the “common” adults. In general, every neighborhood is likely to be walkable (e.g., high residential density and land-use mix), leading to the lack of association with walking duration in Yuncheng. In short, our study reveals that blindly using the findings and policies of walkable neighborhoods from western countries might be ineffective or even counterproductive in smaller Chinese cities because they should be culturally tailored.

A major strength of this study was the use of face-to-face interviews to improve the quality of the questionnaire survey and the depth of discussion. The empirical knowledge, which was gathered from direct interaction with TLS who lived in the Chinese neighborhood environment, is the kernel of this study, as it depicts “real-life” views and clarifies the issues under investigation. This increases confidence in, and provides a more comprehensive understanding of, the key findings. The sample size is small and in a single case, so that the findings cannot be generalized for other cities in China, and this cross-sectional study cannot infer causality. For example, land-use mix might not be significant to promote physical activity in our study, but it might because the variables we adopted, the mixture not reach the threshold, or the quality of land use mix. The findings, however, offer insights to support further larger-scale studies in smaller Chinese cities and reveal that the application of existing policies for walkable neighborhoods adopted from western countries needs to be reconsidered in contemporary China [13,25,31].

This study was an initial exploration and we reduced the length of the questionnaire to improve its practicality in the context of the case study population. For example, the majority of participants lost their patience when the questionnaire was over four pages long. In further questionnaire design, the walking duration could be related to the number of trips and different walking modes (e.g., recreational walking or walking for transport). In addition, we only conducted our study in the central urban area. Further studies could select more and less walkable neighborhoods in order to compare the nuanced results. Future research also should consider aspects of the socio-cultural features (e.g., recreational and educational amenities) and age-related spatial separations when exploring the associations between the neighborhood environment and physical activity.

In spite of the various associations discussed in this study, our strongest recommendation for practitioners of planning, architecture, and landscape is simple: practitioners should recognize that a) increasing land-use mix and high residential density for increasing physical activity might be not applicable in smaller Chinese cities; and b) the benefits of walkability might not be increased equally among different life stages in the same neighborhood environment. Therefore, we have to learn from local patterns of everyday lives, rather than simply implementing a universal strategy and/or design guidance to increase walkability. This initial exploration has enabled us to propose several specific recommendations for practitioners, which could more effectively increase walking/physical activity and then enhance public health in all contexts:

- To recognize that the associations of walking/physical activity with the built environment, walking motivations and sociodemographic characteristics are likely to vary across different life stages, leading to socio-spatial segregation in the same built environment.
- To use age-related and socio-ecological frameworks to plan/design holistic approaches to increasing walkability.
- To understand how “real life” occurs and why walking/physical activity emerges at specific places in smaller Chinese cities.
- To expand definitions of walkability beyond the built environment to involve other impact factors and associations to walking/physical activity (e.g., society and culture).
- To monitor interventions and re-evaluate investments for different life stages, such as providing free government-financed amenities to increase older people’s physical activity and intervening educational or recreational amenities in residential neighborhoods.

## Figures and Tables

**Table 1:** Descriptive analysis of sociodemographic characteristics (*n* = 173).

	Pooled Data		Age Groups						*p*-Value of Difference across TLS
Sociodemographic Characteristics	Yuncheng (All TLS)		Late Adolescents (Aged 18–25)		Young Adults (Aged 26–35)		Middle-Aged Adults (Aged 36–59)		
	(*n* = 173)		(*n* = 48)		(*n* = 68)		(*n* = 57)		
	*n*	%	*n*	%	*n*	%	*n*	%	
*Gender*									
Male	91	52.6	27	56.3	36	52.9	28	49.1	0.765
Female	82	47.4	21	43.8	32	47.1	29	50.9	
*Living with children*	92	53.2	6	12.5	42	61.8	44	77.2	0.000 **
Not living with children	81	46.8	42	87.5	26	38.2	13	22.8	
*Education level*									
High school or below	28	16.2	4	8.3	6	8.8	18	31.6	0.001 **
Junior college	89	51.4	28	58.3	42	61.8	19	33.3	
Bachelor or higher	56	32.4	16	33.3	20	29.4	20	35.1	
*Occupation*									
State worker	43	24.9	4	8.3	21	30.9	18	31.6	0.000 **
Corporate workers	49	28.3	14	29.2	23	33.8	12	21.1	
Self-employee	33	19.1	2	4.2	15	22.1	16	28.1	
Other	48	27.7	28	58.3	9	13.2	11	19.3	
*Income (Yuan)*									
3000 or below	27	15.6	15	31.3	9	13.2	3	5.3	0.010 **
3001–5000	62	35.8	17	35.4	26	38.2	19	33.3	
5001–10,000	65	37.6	12	25.0	27	39.7	26	45.6	
10,001 or above	19	11.0	4	8.3	6	8.8	9	15.8	

** *p* ≤ 0.01 (2-tailed) in Chi-square test or one-way ANOVA. TLS = three life stages.

**Table 2:** Descriptive analysis of household ownership of transports, walking relevance and environmental characteristics (*n* = 173).

Heading	Pooled Data		Age Groups						*p*-Value of Difference across TLS
	Yuncheng (All TLS)		Late Adolescents (Aged 18–25)		Young Adults (Aged 26–35)		Middle-Aged Adults (Aged 36–59)		
	(*n* = 173)		(*n* = 48)		(*n* = 68)		(*n* = 57)		
	Mean	S.D.	Mean	S.D.	Mean	S.D.	Mean	S.D.	
**Household ownership of transports**									
Number of bicycles (0–3)	0.94	0.881	1.06	0.885	0.75	0.780	1.07	0.961	0.069
Number of motorcycles (0–3)	0.90	0.783	0.98	0.838	0.82	0.752	0.93	0.776	0.545
Number of cars (0–3)	0.97	0.758	0.83	0.975	1.01	0.611	1.04	0.706	0.332
**Walking duration**									
Time spent on daily walking (1–5)	2.95	0.871	2.83	0.907	2.79	0.839	3.23	0.824	0.011 *
**Walking preference**									
Do you like walking? (1–3)	2.47	0.728	2.35	0.887	2.53	0.701	2.49	0.601	0.426
**Walking motivation**									
Child walking (1–5)	2.19	1.016	1.71	0.910	2.23	1.04	2.55	0.920	0.000 **
Recreational walking (1–5)	2.47	0.831	2.51	0.914	2.32	0.712	2.61	0.875	0.139
Walking to work (1–5)	2.65	1.274	2.79	1.58	2.57	1.03	2.63	1.26	0.657
Social walking (1–5)	2.85	0.822	3.08	0.919	2.85	0.738	2.65	0.790	0.025 *
**Environmental characteristics**									
Land-use mix (mean of 20 items; 1–5)	3.19	0.658	3.43	0.611	3.16	0.653	3.03	0.656	0.006 **
LUM-daily essential (mean of 7 items; 1–5)	3.72	0.831	3.96	0.755	3.69	0.848	3.56	0.841	0.047 *
LUM-recreation (mean of 5 items; 1–5)	2.67	0.703	2.95	0.700	2.64	0.724	2.48	0.612	0.002 **
LUM-education (mean of 2 items; 1–5)	2.63	0.859	2.89	0.794	2.68	0.922	2.36	0.766	0.006 **
LUM-service (mean of 2 items; 1–5)	2.13	0.707	2.26	0.857	2.07	0.634	2.11	0.646	0.326
Residential density score (355–1775) ^†^	778.1	201.6	729.1	195.0	797.1	183.7	796.7	223.0	0.140
Social quality (mean of 3 items; 1–4)	2.93	0.475	2.85	0.514	2.93	0.467	3.01	0.445	0.209
Street quality (mean of 3 items; 1–4)	2.48	0.564	2.51	0.638	2.47	0.525	2.47	0.553	0.924
Safety (single item; 1–4)	2.97	0.806	3.13	0.815	2.93	0.798	2.88	0.803	0.258

* *p* ≤ 0.05 (2-tailed) in Chi-square test or one-way ANOVA. ** *p* ≤ 0.01 (2-tailed) in Chi-square test or one-way ANOVA. ^†^ Residential density score was calculated by single-family detached, 20*houses 1-3 stories, 35* apartments 4-6 stories, 50* apartments 7-12 stories, 100* apartments 13-26 stories, 150* apartments 27 stories or above. S.D.—standard deviation.

**Table 3:** Multiple linear regression with walking duration (*n* = 173).

	Pooled Data (*n* = 173)			Age Groups								
				Late Adolescents (18–25)			Young Adults (26–35)			Middle-Aged Adults (36–59)		
	Model 1	Model 2	Model 3	Model 1	Model 2	Model 3	Model 1	Model 2	Model 3	Model 1	Model 2	Model 3
**Adjusted R Square**	0.044	0.122	0.245	0.015	0.313	0.428	−0.043	0.162	0.444	0.272	0.360	0.533
**Significance**	0.089	0.002	0.000	0.427	0.025	0.007	0.668	0.062	0.000	0.009	0.003	0.000
Standardized Coefficients												
Beta												
**Sociodemographic characteristics**												
*Gender*												
Female	0.165*	0.108	0.086	0.271	0.267	0.234	0.289	0.229	0.023	−0.102	−0.125	−0.114
Male (reference group)												
*Education level*												
Junior college	−0.347 **	−0.300 **	−0.216 *	−0.637 *	−0.454	−0.440	0.062	−0.053	−0.028	−0.108	0.014	−0.025
Bachelor +	−0.286 *	−0.251 *	−0.108	−0.549	−0.463	−0.451	0.168	0.003	0.089	−0.070	0.030	0.185
High school or lower (reference group)												
*Occupation*												
State worker	0.011	−0.039	−0.094	0.171	0.220	0.225	−0.116	−0.450	−0.441 *	−0.283	−0.394	−0.338
Corporate workers	−0.090	−0.069	−0.069	0.017	−0.023	−0.056	−0.234	−0.595 *	−0.452 *	−0.238	−0.306	−0.215
Self-employee	−0.160	−0.186 *	−0.192 *	0.119	−0.338	−0.521 *	−0.219	−0.537 *	−0.319	−0.346 *	−0.394 *	−0.181
Other (reference group)												
*Income (Yuan)*												
3001–5000	0.167	0.185	0.169	0.123	0.360	0.465 *	0.067	0.265	0.095	0.674 *	0.820 **	0.759 **
5001–10,000	0.229	0.265 *	0.218	0.090	0.087	0.227	0.303	0.601 *	0.325	0.741 *	0.941 **	0.918 **
10,001+	0.065	0.101	0.044	−0.153	−0.035	−0.133	0.045	0.051	−0.077	0.474	0.725 **	0.645 **
3000 or below (reference group)												
*Living with children*												
Living with	−0.036	−0.063	−0.074	−0.156	−0.282	−0.445 *	0.102	0.075	0.249	−0.464 **	−0.592 **	−0.550 **
Not living with children (reference group)												
*Household ownership of transportations*												
No. bicycles	0.071	0.038	0.018	0.052	0.051	−0.053	0.034	−0.038	−0.206	−0.067	−0.141	0.061
No. motorcycles	−0.069	−0.053	−0.045	−0.049	0.079	0.234	0.021	0.093	0.151	−0.011	0.021	−0.027
No. cars	0.083	0.119	0.147	0.250	0.173	0.269	−0.186	−0.035	0.115	0.073	0.073	0.103
Walking preference												
Do you like walking?		0.100	0.132		−0.166	−0.183		0.141	0.183		0.411 **	0.272
Walking motivation												
Recreational walking		0.211 *	0.183 *		0.836 **	0.950 **		0.100	0.215		−0.310	−0.192
Child walking		0.094	0.033		−0.110	−0.182		−0.130	−0.351		0.182	0.254 *
Walking to work		0.062	0.085		−0.160	−0.070		0.484 **	0.342 *		−0.057	0.032
Environmental characteristics												
LUM-recreation			−0.222 **			−0.470 **			−0.388 **			0.189
LUM-education			−0.060			0.094			−0.000			−0.485 **
Social quality			0.311 **			0.100			0.595 **			0.193

* *p* ≤ 0.05 (2-tailed) in Chi-square test or one-way ANOVA. ** *p* ≤ 0.01 (2-tailed) in Chi-square test or one-way ANOVA. No. bicycles—number of bicycles; No. motorcycles—number of motorcycles; No. cars—number of cars.

## Supplementary Materials

The following are available online at https://www.mdpi.com/1660-4601/17/1/237/s1, Table S1: Factor analysis of walking motivation in Yuncheng (*n* = 173), Table S2: Factor analysis of neighborhood quality in Yuncheng (*n* = 173), Table S3: Factor analysis of land-use mix in Yuncheng (*n* = 173), Table S4: Bivariate correlations among walking duration, walking preference, walking motivation, and environmental characteristics in Yuncheng (*n* = 173).
