# Supplementary material for: Exploring the Associations of Walking Behavior with Neighborhood Environments by Different Life Stages: A Cross-Sectional Study in a Smaller Chinese City
Source: Int J Environ Res Public Health. 2019 Dec 28;17(1):237. doi: 10.3390/ijerph17010237 (PMC6982100; doi:10.3390/ijerph17010237)
Supplement: Supplementary file 1 [file ijerph-17-00237-s001.pdf]

Factor analysis

Table S1. Factor analysis of walking motivation in Yuncheng (*n* = 173).

	Component			
	**1**	**2**	**3**	**4**
	**Child Walking**	**Recreational Walking**	**Walking to Work**	**Social Walking**
Cronbach's Alpha Score	0.668	0.628	-	-
(1) Walking with children	0.876			
(2) Walking to school	0.843			
(3) Walking to chat, or play chess and poker		0.894		
(4) Walking to stroll or exercise		0.763		
(5) Walking to work			0.926	
(6) Walking for shopping or dinner				0.967

Extraction Method: Principal Component Analysis. Rotation Method: Varimax with Kaiser Normalization. Kaiser-Meyer-Olkin Measure of Sampling Adequacy: 0.614; Sig.: 0.000.

Table S2. Factor analysis of neighborhood quality in Yuncheng (*n* = 173).

	Component		
	**1**	**2**	**3**
	**Social Quality**	**Street Quality**	**Safety**
Cronbach's Alpha Score	0.556	0.509	-
(1) There are trees along the streets in my neighborhood	0.718		
(2) There are many interesting things to look at while walking in my neighborhood	0.705		
(3) I often meet familiar people when I am walking around	0.691		
(4) People thrown rubbish arbitrarily in my neighborhood		0.773	
(5) There is no parking problem around my neighborhood		0.706	
(6) There are attractive buildings/homes in my neighborhood		0.644	
(7) The crime rate in my neighborhood makes it unsafe to go on walks at night			0.954

Extraction Method: Principal Component Analysis. Rotation Method: Varimax with Kaiser Normalization. Kaiser-Meyer-Olkin Measure of Sampling Adequacy: 0.585; Sig.: 0.000.

Table S3. Factor analysis of land-use mix in Yuncheng (*n* = 173).

	Component			
	**1**	**2**	**3**	**4**
	**Daily Essential**	**Recreation**	**Education**	**Service**
Cronbach's Alpha Score	0.917	0.782	0.535	0.500
(1) Fruit/vegetable vending	0.759			
(2) Small grocery store	0.747			
(3) Fruit/vegetable shop	0.743			
(4) Restaurant	0.728			
(5) Supermarket	0.704			
(6) Snacks/breakfast (vending)	0.686			
(7) Salon/barber shop	0.633			
(8) Bus station	0.621			
(9) Park/square	0.607			
(10) Laundry/dry cleaner	0.554			
(11) Pharmacy/drug store	0.545			
(12) Gym or fitness facility		0.824		
(13) Karaoke bar		0.773		
(14) Cinema		0.625		
(15) Bath center		0.554		

(16) Internet bar	0.538			
(17) Other schools		0.697		
(18) Book store		0.538		
(19) Library			0.781	
(20) Post office			0.663	

Extraction Method: Principal Component Analysis. Rotation Method: Varimax with Kaiser Normalization. Kaiser-Meyer-Olkin Measure of Sampling Adequacy: 0.894; Sig.: 0.000.

Bivariate analysis

Supplementary Table 4 shows the bivariate correlations between walking duration, walking preference, walking motivation and environmental characteristics. All the bivariate correlations are further divided into TLS (a = late adolescents, b = young adults and c = middle-aged adults). Given the research aim, the results focus on the correlations of walking duration to other sub-scales.

Table S4. Bivariate correlations among walking duration, walking preference, walking motivation and environmental characteristics in Yuncheng (*n* = 173).

		1 Walking Duration	2 Walking Preference	3 Recreational Walking	4 Child Walking	5 Walking to Work	6 Social Walking
(1) Walking duration		1					
(2) Walking preference	All	0.195 *	1				
	a	0.154	1				
	b	0.188	1				
	c	0.274 *	1				
(3) Recreational walking	All	0.303 **	0.330 **	1			
	a	0.400 **	0.310 *	1			
	b	0.261 *	0.273 *	1			
	c	0.201	0.477 **	1			
(4) Child walking	All	0.159 *	0.103	0.211 **	1		
	a	0.146	0.065	0.445 **	1		
	b	0.183	0.088	−0.003	1		
	c	−0.004	0.098	0.265 *	1		
(5) Walking to work	All	0.141	0.377 **	0.343 **	0.125	1	
	a	0.035	0.523 **	0.406 **	0.156	1	
	b	0.399 **	0.256 *	0.504 **	0.295 *	1	
	c	0.031	0.337 *	0.149	0.002	1	
(6) Social walking	All	0.030	0.148	0.239 **	0.069	0.311 **	1
	a	0.145	0.355 *	0.290 *	0.081	0.524 **	1
	b	−0.001	0.037	0.203	0.005	0.408 **	1
	c	0.070	0.069	0.274 *	0.394 **	−0.042	1
(7) Land-use mix (mean of 20 items)	All	−0.106	0.142	0.085	−0.167 *	0.150 *	0.113
	a	−0.150	0.045	−0.013	−0.069	0.258	0.105
	b	0.003	0.179	0.121	−0.104	0.070	0.184
	c	0.097	0.304 *	0.158	−0.108	0.105	−0.094
(8) LUM-daily essential	All	−0.061	0.110	0.017	−0.174 *	0.111	0.060
	a	−0.194	−0.015	−0.138	−0.139	0.169	−0.028
	b	0.071	0.129	0.026	−0.060	0.045	0.168
	c	−0.032	0.297 *	0.142	−0.193	0.104	−0.096
(9) LUM-recreation	All	−0.195 *	0.080	0.124	−0.182 *	0.107	0.152 *
	a	−0.094	0.029	0.097	−0.020	0.263	0.199
	b	−0.227	0.103	0.194	−0.248 *	−0.036	0.162
	c	−0.151	0.220	0.114	0.040	0.053	−0.073
(10) LUM-education	All	−0.134	0.146	0.102	−0.067	0.167 *	0.148
	a	0.002	0.225	0.148	0.071	0.285 *	0.320 *
	b	0.059	0.217	0.155	−0.052	0.163	0.095
	c	−0.401 **	0.017	0.076	0.055	0.047	−0.098
(11) LUM-service	All	0.059	0.240 **	0.248 **	0.108	0.213 **	0.110
	a	0.057	0.128	0.241	0.161	0.221	0.188
	b	0.068	0.289 *	0.275 *	0.158	0.262 *	0.053
	c	0.071	0.416 **	0.225	0.126	0.1360	0.021
(12) Residential density	All	0.085	−0.042	0.013	0.079	−0.004	−0.052

	a	0.147	0.079	0.142	−0.117	−0.092	0.039
	b	0.194	0.022	0.004	0.192	0.125	0.070
	c	−0.098	−0.302 *	−0.063	−0.024	0.001	−0.172
(13) Social quality	All	0.324 **	0.047	0.217 **	0.165 *	0.060	0.108
	a	0.081	−0.050	0.358 *	0.213	0.012	0.028
	b	0.443 **	−0.001	0.048	0.244 *	0.234	0.199
	c	0.382 **	0.223	0.241	−0.111	−0.035	0.198
(14) Street quality	All	−0.107	−0.177 *	−0.097	−0.118	−0.134	−0.120
	a	−0.268	−0.111	−0.149	−0.241	−0.160	−0.255
	b	0.108	−0.112	−0.080	−0.038	−0.023	−0.090
	c	−0.189	−0.355 **	−0.068	−0.103	−0.215	−0.022
(15) Safety	All	−0.118	0.018	0.055	−0.148	−0.142	−0.017
	a	−0.058	0.143	0.184	0.007	−0.045	−0.043
	b	−0.068	−0.009	−0.011	−0.213	−0.276 *	−0.044
	c	−0.200	−0.058	0.006	−0.088	−0.151	−0.041

**. $p \leq 0.01$ (2-tailed) in Chi-square test or One-way ANOVA. *. $p \leq 0.05$ (2-tailed) in Chi-square test or One-way ANOVA. All = pooled data; a = late adolescence (aged 18–25); b = young adults (aged 26–35); c = middle-aged adults (aged 36–59).
